# A First Case Report of Subependymoma in *PTPN11* Mutation-Associated Noonan Syndrome

**DOI:** 10.1155/2019/6091059

**Published:** 2019-09-16

**Authors:** Boonchai Boonyawat, Mongkon Charoenpitakchai, Piradee Suwanpakdee

**Affiliations:** ^1^Division of Genetics, Department of Pediatrics, Phramongkutklao Hospital and Phramongkutklao College of Medicine, Bangkok, Thailand; ^2^Department of Pathology, Phramongkutklao Hospital and Phramongkutklao College of Medicine, Bangkok, Thailand; ^3^Division of Neurology, Department of Pediatrics, Phramongkutklao Hospital and Phramongkutklao College of Medicine, Bangkok, Thailand

## Abstract

Noonan syndrome (NS) is an autosomal dominant disorder in some cases caused by *PTPN11* mutations. Since somatic mutations in *PTPN11* are seen in several tumor types, NS which causes germline *PTPN11* mutations are also increase the risk of hematologic malignancies and brain solid tumors. However, the report of brain tumors in Noonan syndrome remains rather rare. Here, we report the first case of an 11-year-old Thai boy with Noonan syndrome who presented with symptoms related to hydrocephalus secondary to subependymoma in the fourth ventricle, and *PTPN11* mutation was identified in this patient.

## 1. Introduction

Noonan syndrome (NS, OMIM 163950) is an autosomal dominant multiple congenital anomaly syndrome, which was first described by Jacqueline Noonan in 1968 [1]. The estimated prevalence is between 1 : 1,000 and 1 : 2,500 live births [2]. NS is characterized by short stature, distinctive facial dysmorphism, congenital heart defects, and a variable degree of developmental delay. Heterozygous activating mutations of the protein-tyrosine phosphatase, nonreceptor-type 11 genes (*PTPN11*, OMIM 176876) is the main cause of NS, which was elucidated by Tartaglia et al. in 2001 [3, 4]. The *PTPN11* gene encodes the nonreceptor protein tyrosine phosphatase (PTP) SHP-2 which is one of the proteins in the RAS-MAPK signaling pathway involved in the process of cell growth and differentiation.

Somatic mutations of *PTPN11* have been identified as the main cause of juvenile myelomonocytic leukemia (JMML) and in a small percentage of myelodysplastic syndrome (MDS) and acute myeloid leukemia (AML) [5]. Similarly, solid tumors have also been associated with somatic *PTPN11* mutations [6]. Thus, individuals with NS are also increased risk of cancer, in particular, hematologic malignancies and, less frequently, solid tumors including central nervous system (CNS) tumors [7]. Among CNS tumors, previous studies reported the low-grade glial and glioneural tumors, primarily dysembryoplastic neuroepithelial tumors (DNET) and low-grade gliomas [8, 9] but so far, the subependymoma has never been reported.

Herein, we report the first case of an 11-year-old Thai boy with *PTPN11* mutation-associated NS who developed subependymoma (WHO grade I) in Phramongkutklao hospital, Thailand.

## 2. Patients and Methods

### 2.1. Patients

A male patient was born at term with an uneventful pregnancy. His birth weight was 3,190 g (P50) and his birth length was 49 cm (P25). Physical examination at birth revealed dysmorphic facial features including left eye ptosis and triangular face, webbed neck, mild pectus deformity, and widely spaced nipples. The atrial septal defect was detected at the age of 9 months and surgical closure was performed at the 2 years of age. Short stature was detected at the age of 5 years. Owning to facial dysmorphism, a congenital heart defect, and short stature, he was diagnosed with Noonan syndrome. Growth hormone (GH) study revealed partial GH deficiency and GH treatment has been started since then.

**Figure 1:**
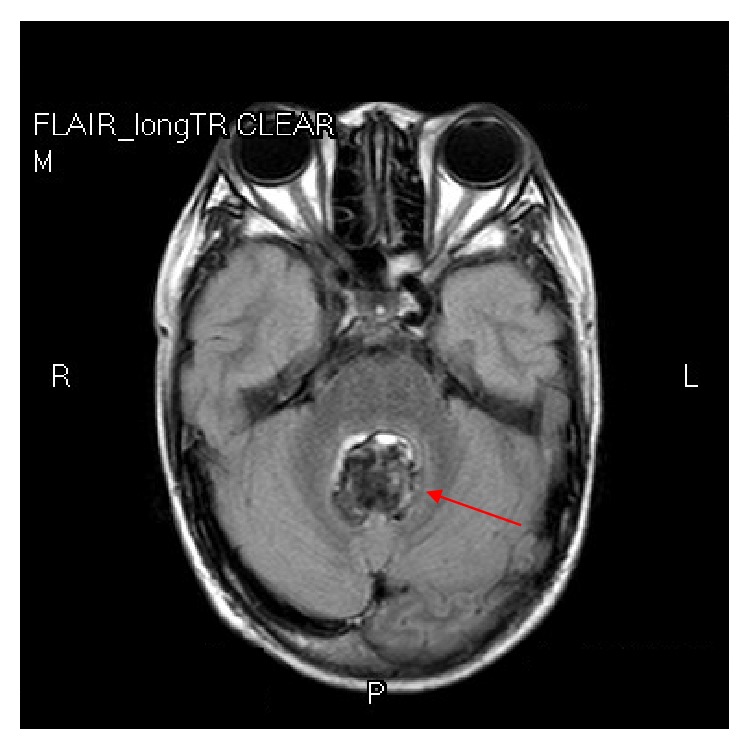
Magnetic resonance imaging (FLAIR, axial view) showing a lobulated, well-defined intraventricular mass (arrow) with no paraventricular extension attaching the fourth ventricular floor.

**Figure 2:**
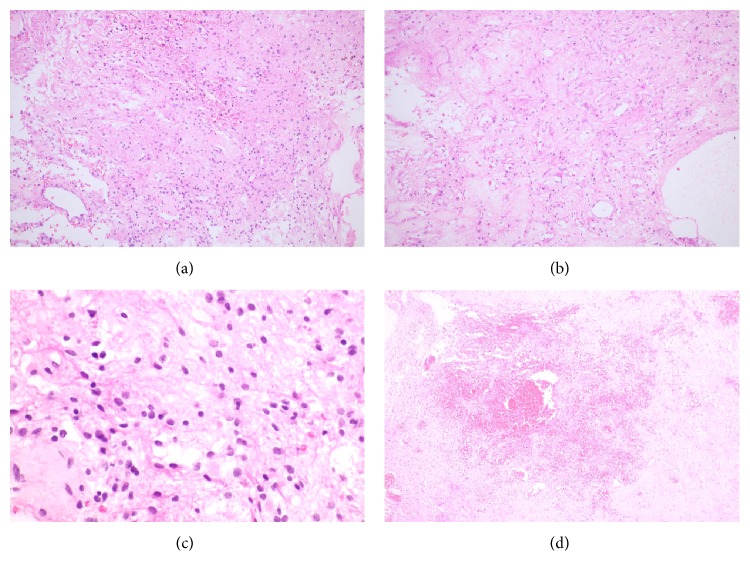
The mass showed delicately fibrillar stroma with clusters of nuclei (a, H&E 100x) and microcystic change (b, H&E 100x). Tumor nuclei are round to oval and isometric with salt and pepper chromatin (c, H&E 400x). Area of tumor hemorrhage (d, H&E 100x).

At 11 years of age, he presented with one week of progressive headache and repeated vomiting, especially in the morning. Neither history of weakness nor seizures were noted. Neurological examination revealed only bilateral papilledema, the rest of the detailed neurological examination was normal. Magnetic resonance imaging (MRI) of the brain revealed a lobulated, well-defined intraventricular mass with no paraventricular extension attaching the fourth ventricular floor causing obstructive hydrocephalus (Figure 1). He underwent craniotomy and complete macroscopic removal of the tumor. Postoperative tumor histopathology revealed characteristic features of subependymoma with delicately fibrillar stroma with clusters of nuclei (Figure 2(a)) and microcystic change (Figure 2(b)). Tumor nuclei are round to oval and isometric with salt and pepper chromatin (Figure 2(c)). No mitosis was seen. Focal area of increased vasculature with sclerotic change as well as hemorrhage and calcification are also observed (Figure 2(d)). His headache resolved postoperatively and no radiological evidence of recurrence was seen at 7 years of follow-up.

### 2.2. Mutation Analysis of the *PTPN11* Gene and Result

After informed consent was obtained from the patient and the parents, genomic DNA was extracted from peripheral blood lymphocytes. All 15 coding exons and exon-intron boundaries of *PTPN11* were amplified by PCR as described previously(I4). All PCR products were directly sequenced in both directions.

A heterozygous missense; c.922A>G (p.Asn308Asp), mutation of *PTPN11* has been identified in the patient DNA. This mutation was not identified in both paternal and maternal DNA suggesting *de novo* mutation in the patient.

## 3. Discussion

Noonan syndrome (NS) is an autosomal dominant disorder which was characterized by characteristic facial features, short stature, and congenital cardiac defects. Missense mutations in *PTPN11* accounted for 31%–60% of NS cases [10]. These mutations were demonstrated to be gain-of-function resulting in excessive SHP-2 activity. Generally, overall children with NS have an estimated 8.1-fold increased risk of childhood cancers, whereas individuals with *PTPN11* mutation-associated NS have approximately 3.5-fold increased risk of all cancers including both hematologic and solid malignancies compared with the general population [11, 12]. Hematologic cancers widely accepted to be associated with NS include juvenile myelomonocytic leukemia (JMML), acute myelogenous leukemia (AML) and acute lymphoblastic leukemia (ALL) [2, 5]. Solid tumors including rhabdomyosarcoma, neuroblastoma, and CNS tumors were also reported [11, 12]. The majority of brain tumors associated with NS are low-grade glial and glioneural tumors, primarily dysembryoplastic neuroepithelial tumors (DNET) and low-grade gliomas [8, 9].

To date, a total of 27 cases of brain tumor associated with NS have been reported in the literature [8, 9]. The mean age at the time of brain tumor diagnosis for all patients was 16 years (ranging from 6–37 years). Majority of the patients were male. Approximately 80 percent of cases were caused by a heterozygous missense mutation in *PTPN11*, whereas, NS was diagnosed by clinical phenotypes only in the remaining (20%) of the patients. As in our patient who was a boy with NS, subependymoma (WHO grade I) was detected at the age of 11 years. Mutation analysis of *PTPN11* revealed a previously reported heterozygous missense p.Asn308Asp mutation [10]. This mutation has been previously identified in 3 NS associated CNS tumor patients. The first patient was a 6-year-old girl with dysembryoplastic neuroepithelial tumor (DNET), which is a rare CNS neoplasm [11]. The second patient was a 14-year-old female with anaplastic astrocytoma at the left brainstem and cerebellar peduncle [8]. The last patient was a 16-year-old male with DNET at left temporal and frontal lobe, and right thalamus [9]. Our study was the first report of subependymoma in the fourth ventricle in NS patients. Subependymoma is an extremely rare benign tumor accounting for less than 1% of all intracranial tumors and slow progression of symptoms in nature [13, 14]. Contrary to our patient, he developed symptoms related to hydrocephalus at the early age of presentation. This finding may explain from the moderate size of the tumor and location in the fourth ventricle which easily obstruct the cerebrospinal fluid (CSF) pathway. Common imaging characteristics of subependymoma include a lobulated, well-defined intraventricular mass with no paraventricular extension which was similar to our case [15]. The standard treatment is complete tumor resection which influenced the good prognosis. Our case confirmed this statement; he underwent total tumor resection without post-operative complication and achieved an excellent surgical outcome. Post-operative MRI brain was performed annually, no evidence of tumor recurrence was seen at 7 years of follow-up.

## 4. Conclusion

Our study demonstrates the first case of symptomatic subependymoma in *PTPN11* mutation-associated NS. Even though, there is no correlation between the specific mutation in *PTPN11* and the occurrence of cancer, approximately 15 percent of brain tumors in *PTPN11* mutation-associated NS were caused by heterozygous of p.Asn308Asp mutation in *PTPN11*. A high index of suspicion is required to explore CNS tumors in Noonan syndrome with *PTPN11* mutation patients who present with progressive headache as well as before starting GH therapy.
